# Association of Angiotensin Converting Enzyme gene Polymorphism and Indian Army Triathletes Performance

**DOI:** 10.5812/asjsm.34855

**Published:** 2010-09

**Authors:** Shweta Shenoy, Suparna Tandon, Jaspal Sandhu, Amarjeet Singh Bhanwer

**Affiliations:** Department of Sports Medicine & Physiotherapy, Guru Nanak Dev University, Amritsar, Punjab, India

**Keywords:** ACE, Polymorphism Genetic, Angiotensin converting enzyme, Physical fitness, Genotyping

## Abstract

**Purpose:**

It is well known that the effects of exercise training gives inter individual differences which might be due to genetic diversity. This study aims to explore the probable relation between angiotensin converting enzyme (ACE) alleles and physical fitness parameters in elite athletes.

**Methods:**

Twenty-nine national level Indian Army Triathletes who volunteered for the study were taken as subjects and 101 healthy age matched control group subjects were taken for comparison of genotype frequencies. The following parameters were checked in triathletes: blood pressure, body mass index, VO_2 max_, muscular endurance, flexibility and power. DNA was extracted from blood using standard phenol-chloroform method. Genotyping was done using PCR specific for ACE (I/D) polymorphism, followed by agarose gel electrophoresis method. Variation of the parameters among different genotypes was compared. Genotype frequencies of triathletes were compared with the control group as well.

**Results:**

No difference was observed between fitness parameters of three genotype groups’ triathletes, while the frequency of I allele was found to be very high in triathletes compared with the control group.

**Conclusion:**

It can be concluded that there might be a positive association between I allele of ACE gene and endurance.

## INTRODUCTION

It is well known that the effects of exercise training gives inter individual differences^[1]^. This individual variability in exercise responses has been described as a normal biological phenomenon that may reflect genetic diversity^[2]^. One of such genetic factors affecting response to training is angiotensin converting enzyme gene polymorphism which has been widely studied ^[3, 4]^. Angiotensin-converting enzyme (ACE, EC 3.4.15.1, dipeptidyl carboxypeptidase) is associated with the regulation of blood pressure and maintenance of salt and water homeostasis of the body^[5]^. Renin which is secreted from juxtaglomerular cells in the kidney acts on angiotensinogen and converts it into angiotensin I^[6]^. Angiotensin converting enzyme (ACE) converts angiotensin I into angiotensin II, which is the final active material^[6]^. Such physiological response is predominantly mediated by Angiotensin II specific receptors (AT_1_ and AT_2_), located on the cellular surface^[7]^.

ACE gene (21 Kbp) is located on the chromosome 17 q23 and is composed of 26 exons^[8]^. There is a 287 bp insertion (I) / deletion (D) polymorphism in intron 16 of ACE gene that occurs commonly and accounts for a substantial portion of the variance in serum ACE levels^[9]^. D allele has been associated with a higher ACE activity in the serum and tissue than the I allele^[9]^.

Some studies have associated I allele with greater endurance^[3, 10–12]^ and D allele with greater strength^[13, 14]^. Yet, there have been other studies which have found no association between these variables^[15–17]^.

Several studies have been done in various parts of the world but not on Indian population. We have made an attempt to bridge this gap and contribute to the worldwide data being generated on the association between ACE polymorphism and physical fitness phenotypes. The purpose of this study was to find if any association exists between the elite endurance athlete status and ACE polymorphism in Indian population. We also wanted to find whether any difference exists between the three genotype groups of the endurance athletes concerning the physical fitness parameters, such as maximum volume of oxygen uptake (VO_2__max_), power, muscular endurance, flexibility and body mass index (BMI).

## METHODS AND SUBJECTS

Twenty-nine national level Indian army Triathletes who volunteered for the study underwent measurements of blood pressure, BMI, VO_2_ max, muscular endurance, power and flexibility. One-hundred one healthy age matched control subjects were taken for comparison of genotype frequencies.

**Sampling:** Sample collection was done after obtaining written informed consent and about 2ml of blood was collected in vials containing EDTA and was transported to laboratory on ice. The study was conducted in accordance with Declaration of Helsinki after approval of local ethics committee.

**Determining genotype**: DNA was extracted from blood by using standard phenol-chloroform method^[18]^. DNA quantification was done by agarose gel electrophoresis. Desired region of DNA was amplified by PCR to study ACE insertion/deletion polymorphism. Presence of 190 bp fragment indicated the presence of Deletion (D) allele and that of a 490 bp fragment indicated the presence of Insertion (I) allele. The individuals homozygous for the insertion allele were designated as II, heterozygous as ID and those homozygous for deletion allele were designated as DD. In order to increase the specificity of DD genotyping, all samples identified as DD after initial amplification were reconfirmed with an insertion-specific primer pair.

The presence of insertion sequence was revealed by the amplification of a 275 bp fragment, while DD homozygotes failed to be amplified due to the lack of annealing site^[19]^. The genotypes of all subjects were recorded.

**Measurement of VO_2 max_:** VO_2 max_ measurements were taken using Queen's college step test^[20]^.

*Procedure followed for step test*: A 16.25 inches high stepper was used. Subjects had to step up and down for 3 min at a rate of 24 steps per min. Rate was regulated using a metronome. The subject immediately stopped on completion of the test, and the heart beats were counted for 15 seconds from 5-20 seconds of recovery and recorded. The following formula was used to calculate VO_2__max_:

$$VO_{2}\max(\mathrm{ml}/\mathrm{kg}/\min)=\mathrm{111}\mathrm{.33}-(0.42x\mathrm{step}-\mathrm{test}\mathrm{pulse}\mathrm{rate}\left[\mathrm{beats}/\min\right])$$

**Measurement of muscular endurance:** Muscular endurance was assessed using one minute push-up and curl-up test^[21]^.

*Procedure for push-up test:* Subjects performed this test in the standard ‘on toes’ position. The subject lowered the body until the chin touched the mat (It was instructed that abdomen should not touch the mat). Subject was reminded to keep his back straight at all times and to push up to a straight-arm position. The maximum number of push-ups performed consecutively without rest was counted.

*Procedure for Curl-ups:* The subject assumed a supine position with knees bent so that the heels are positioned approximately 18 inches from the buttocks. The arms were held at the side, with a strip of masking tape placed on the floor at the fingertips. A second strip of tape was placed exactly 12 cm beyond the first strip (towards heels). The subject performed slow, controlled curl-ups, lifting the inferior border of scapula off the mat and returning to the start position after each repetition. To successfully complete a repetition, the subject had to touch the fingertips to the second strip of tape, with the trunk making a 30-degree angle with the mat and the low back flattened before each curl-up. At the ‘go’ signal, the subject performed as many curl-ups as possible in 1 min.

**Measurement of muscle strength and power:** Strength of upper body was assessed using push-ups. Vertical jump was used to assess lower body strength.

Harman formula was used to convert vertical jump scores into peak power and average power.

*Procedure:* A measuring tape and chalk for marking the wall was used. The athlete stood with side on to a wall and reached up with the dominant hand closest to the wall. Keeping the feet together and flat on the ground, the point of the fingertips was marked and recorded as the ‘standing reach’. The athlete then performed the vertical jump using the countermovement jump technique described by Adams^[22]^. Each subject was then given three opportunities to jump. Shuffling of feet or steps was not allowed. The difference between the standing reach and the higher jump to the nearest 1cm was recorded as their vertical jump. The difference in distances between the standing reach height and the jump height was the score. The best of three attempts was recorded. Later jump height was converted into a power score.

*Vertical Jump Power Calculation:* We have used the Harman Formula. Harman et al^[23]^ established equations for both peak and average power through multiple regression procedures. The two equations are listed below:

Peak power(W)=61.9jumpheight(cm)+36.0bodymass(kg)+1,822

Averagepower(W)=21.2jumpheight(cm)+23.0bodymass(kg)-1,393

**Measurement of flexibility:** Flexibility was assessed using modified sit and reach test^[24]^.

This test measures the flexibility of the lower back and hamstring muscles.

*Procedure:* Three trials were given following a warm-up. The test involved sitting on the floor with back and head against a wall, legs fully extended, with the bottoms of the feet (shoes off) against the sit-and-reach box. Hands were placed on top of each other, stretching the arms forward while the head and back kept against the wall. The distance from the fingertips to the box edge was measured with a yardstick. This represented the zero, or starting point. Subject was asked to slowly bend forward and reach as far as possible, sliding the fingers along the yardstick; the final position was held for 2 seconds. Total distance reached to the nearest ¼ inch represented the final score.

**BMI:** BMI was measured using height and weight.

$$\mathrm{BMI}=\mathrm{weight}(\mathrm{in}\mathrm{kgs})/{\left[\mathrm{height}(\mathrm{inmeters})\right]}^{2}$$

**Population studied and sample size:** The study subjects were volunteers, 29 male national level Indian army Triathletes with their age ranging from 20 to 25 years (after permission of the authorities) and 101 healthy aged matched subjects as a control group (for comparing genotype frequencies). The sample has been selected from Triathalon that is an event which requires very high endurance. Hence, we wish to study the relation between physical fitness and ACE polymorphism. The subjects belonged to heterogenous genotype groups, while being exposed to similar training and food.

SPSS version 17 was used for analysis of the data. Levene's test was applied to check the homogeneity of variance. One- way ANOVA was used to compare all variables including BMI, VO_2__max_, peak power, average power, number of curl-ups, number of push-ups and score on sit and reach test between the three groups. Consequently, a Post Hoc Tukey's HSD Test was applied. The two groups (triathletes and controls) were compared according to allele and genotype frequencies by using χ^2^ analysis.

## RESULTS

The results obtained after analysis revealed that there was no statistically significant difference between the three genotype groups in any of the parameters measured (*P*>0.05) (Table 1).

**Table 1:** Analysis of variance among the genotypes through One-way ANOVA of various fitness parameters of triathletes

Variables		Sum of Squares	df	Mean Square	F	*P* Value
BMI*	**Between Groups**	5.11	2	2.55	0.87	0.4
	**Within Groups**	75.75	26	2.91		
	**Total**	80.86	28			
**VO**_2 max_**(ml/kg/min)**	**Between Groups**	24.50	2	12.24	0.33	0.7
	**Within Groups**	961.50	26	36.98		
	**Total**	985.94	28			
**Peak power (watts)**	**Between Groups**	70383.50	2	35191.74	0.13	0.9
	**Within Groups**	6780432.81	26	260785.88		
	**Total**	6850816.31	28			
**Average power (watts)**	**Between Groups**	19703.03	2	9851.51	0.25	0.8
	**Within Groups**	1027004.48	26	39500.17		
	**Total**	1046707.51	28			
Curl-ups (n**)	**Between Groups**	21.90	2	10.95	0.45	0.6
	**Within Groups**	637.41	26	24.52		
	**Total**	659.31	28			
**Push-ups (n)**	**Between Groups**	49.13	2	24.57	0.26	0.8
	**Within Groups**	2432.66	26	93.56		
	**Total**	2481.79	28			
**Sit & reach (cms)**	**Between Groups**	3.97	2	1.99	0.04	0.9
	**Within Groups**	1193.73	26	45.91		
	**Total**	1197.70	28			

* BMI: Body mass index

** n = Number

Distributions of I and D alleles and ACE genotypes are given in Table 2. I allele was significantly increased in the triathletes (χ^2^ =5.36, df:1, *P=*0.02). The same trend was found in the genotypic distributions, as triathletes showed an excess of the II genotype (χ^2^ =6.50, df:2, *P=*0.02). Distribution of I and D alleles and their genotypes for the control subjects are listed in Table 2.

**Table 2:** Frequency of the angiotensin- converting enzyme (ACE) insertion (I), and deletion (D) alleles and their genotypes in the two populations

Group	ACE allele		ACE genotype		
	I	D	II	ID	DD
**Triathletes** (n=29)	41 (85%)	7 (15%)	14 (48%)	13 (45%)	2 (7%)
**Controls** (n=101)	106 (52%)	96 (48%)	26 (0.26)	54 (0.53)	21 (21%)

n=Number

Genotype frequencies were in Hardy Weinberg equilibrium in both triathletes and controls, making selection bias less likely. Our results indicate I allele dominance in triathletes compared with the control group. On the other hand, no statistically significant difference was found between the three genotypes (II, ID, DD) in any of the physical fitness parameters measured.

## DISCUSSION

**ACE and VO_2 max_:** There was no statistically significant difference between the VO_2 max_ of triathletes of the three genotype groups (Table 1) while a significant higher frequency of I allele was observed among triathletes compared with the control group (Table 2). This may suggest some sorts of associations between ACE I allele and endurance. Nonetheless, it is not likely to be definite considering lack of differences between VO_2 max_ of three genotypes triathletes. The sample size of our study is also too small to be able to make such a definite assumption. Various studies have suggested an association between ACE I/D polymorphism and fitness parameters. A significant excess of II and ID ACE genotypes was demonstrated in a study involving 25 elite high-altitude British mountaineers^[3]^. Moreover, a positive association was found between I allele and endurance performance among British Olympic standard runners ^[25]^. In another study done on postmenopausal women ACE II genotype carriers had a greater VO_2__max_ than ACE ID or ACE DD genotype carrier^[26]^. Montogmery et al.^[3]^ also reported that I allele is associated with improved endurance capability. Hypoxia induced increased ventilation was shown to be significantly greater among type II genotype carriers^[12]^. Tsianos and colleagues^[27]^ investigated the frequency of the ACE I/D polymorphism among 35 elite endurance swimmers. They found an excess of I allele in swimmers participating in 25-km races requiring more endurance and a significant excess of D allele among subjects competing in 1- to 10-km races, which may require more strength or power. This may suggest a relation between duration of event and I/D polymorphism, with I allele favouring long duration events requiring endurance.

Other recent investigations^[28, 17, 26]^ have failed to show effects of ACE genotype on VO_2 max_ or clustering of ACE genotype II in elite athletes participating in sports with high aerobic fitness demands^[15, 16, 29]^. Additionally, a recent study by Bouchard et al. found no linkage between chromosome 17genetic markers, location of the ACE gene, and either baseline VO_2 max_ or the VO_2 max_ response to training^[30]^.

The effect of ACE genotype may be eclipsed in young adults by the importance of other biological factors, such as the hormonal environment^[28]^. This may explain different responses shown by different studied population groups. Furthermore, although VO_2 max_ is clearly important for aerobic events performance, it only accounts for 80-90% of the variance in subjects’ running times, while lactate threshold and running economy account for most of the rest^[31]^. This may explain why no association was found in some studies.

Researches which have linked D allele with endurance are in contrast to our study ^[32, 33]^. Overall, the preponderance of evidence suggests that some sorts of associations exist between ACE genotypes and endurance. Larger intervention based studies on various homogenous population groups are required to evaluate, validate and consolidate such claims.

**ACE and Strength or Power:** In our study no association was found regarding peak power and average power between three genotypes (Table 1). There have been studies which reported greater strength gains with D allele. Folland et al.^[13]^ conducted a study on 33 healthy males involving 9 weeks of quadriceps strength training to examine the effect of ACE polymorphism on physical performance and finding greater strength gains in subjects with D- allele. Williams et al. in their study found D allele to be associated with isometric and isokinetic quadriceps muscle strength^[14]^. Furthermore, D allele has recently been associated with left ventricular hypertrophy in response to physical training^[34]^. In contrast, Woods et al.^[35]^ in a study on post- menopausal women found that I allele was associated with greater strength gains. In another study ACE ID genotype was found to be associated with the contralateral effects of unilateral resistance training^[36]^.

It is interesting to know that angiotensin II may be important in blood flow redirection from type I muscle fibers to type II fibers^[37]^ that are favoured in power performance. On the contrary our study showed no relation between ACE polymorphism and power. According to the results it can be inferred that either there is no relation or the statistical power of the study is not adequate to detect it. Results may also be confounded by the fact that studied athletes belonged to a high endurance sport.

**ACE and Muscular endurance:** The results obtained in our study showed no significant association between number of performed curl-ups and number of push-ups and any genotype group (Table 1). Though rennin- angiotensin system is active in skeletal muscles, the quantifying muscular endurance parameters failed to show an association with any genotype. This finding is consistent with the results obtained by Sonna et al.^[28]^ who employed similar tests in their study on US army recruits.

**ACE and Flexibility:** No statistically significant association has been found between genotype groups and flexibility (Table 1). This is the first study which has attempted to find an association between these variables.

**ACE and BMI:** We found no statistically significant difference between the genotypes in terms of BMI (Table 1), although some researchers have reported an association between a higher body fat percentage and I allele due to its anabolic effect and better muscular efficiency. Montgomery and colleagues^[38]^ investigated the metabolic effects of the human local renin-angiotensin system within subjects involved in an intensive exercise program and showed that those with II genotype had a greater fat mass anabolic response than did those with either ID or DD genotypes. This obtained data contradicts the findings of Katsuya and colleagues^[39]^, who found an association between the ACE I allele and an increased body mass index.

A significant strength of our study was the fact that a healthy age matched control group was taken for comparison of genotype frequencies. Besides, high motivation levels of army triathletes ensured that they performed their best on all the tests.

One of the limitations of our study was the fact that a heterogenous population was enrolled. Vast genetic dissimilarity can be considered as a potential confounding factor of the findings. Due to practical limitations field tests, which are not as sensitive as direct lab tests, were employed for measuring various physical fitness parameters. However, as direct lab tests are more sensitive, they should be used in future studies to improve the reliability of obtained results.

Furthermore, the very small sample size of triathletes which was available for the present study is not enough to generalise the findings. A larger study done at different centres across the country can take the advantages of a small sample of a homogenous group and yet the combined result would be applicable for a large heterogeneous population. Similar studies on different population groups in terms of age, physical fitness and disease profile can help in better understanding of the role of ACE in physical performance.

Since a positive association is found between genotype and athletic ability, we recommend a larger longitudinal study to screen the children with athletic ability and study their response to training and its association with genetic polymorphism.

## CONCLUSION

Based on the findings of this study it can be concluded that there is an association between I allele of ACE and endurance. There may be separate and complementary actions of I and D alleles. The finding of allele I dominance in triathletic group, and even a suggestion of II homozygote advantage is consistent with this idea.

We also conclude that there is no association between ACE polymorphism and power, muscular endurance, flexibility and BMI in this group of national level Indian army triathletes.
